# Current evidence and prevalence of dementia care in East Africa‐Uganda a systematic review

**DOI:** 10.1002/alz.083767

**Published:** 2025-01-09

**Authors:** Waiswa Moses, Wandera Simon, Namuli Melinda, Kalsonid Davidson

**Affiliations:** ^1^ uganda Development And Health Associates, Iganga Uganda; ^2^ iganga Referral Hospital, Iganga, Uganda Land Uganda; ^3^ makerere University, Kampala, Uganda Land Uganda; ^4^ case Clinic For Dementia, Kampala, Uganda Land Uganda

## Abstract

**Background:**

Uganda has an increasing population of older persons who require special attention to reduce the burden of dementia. With the growing prevalence of dementia worldwide, two third of the people with dementia are projected to be from the de‐eloping countries by 2050. Few studies have been conducted on the prevalence of dementia and its association with central nervous system (CNS) infections among older persons in African settings, particularly in Uganda.

**Methods:**

The study was conducted in March 2022 among 434 older persons aged 50 and above years who were selected by multistage sampling. Data were collected using an inter‐viewer administered questionnaire supplemented with information from participant’s medical records and a brief community screening instrument for dementia. The instrument classifies dementia into unlikely, probable or possible dementia. Data were entered in duplicate into EpiData version 3.0, then transferred to statistical package for social sciences (SPSS) version 23 for statistical analysis.

**Results:**

Our study found almost one in four (23%) of the older persons in tororo district eastern part of Uganda were suffering from probable or possible dementia. Our study further found that older persons with a positive history of central nervous system infections (CNS) had nearly five times higher odds of having probable or possible dementia compared to their counterparts (cOR: 4,5: 2.76‐7.23; p< 0.001). Being in advanced age of 70+ years (aOR: 2.6; 5:4; 1.4 ‐20.5; p = 0.013), and chronic headache (aOR: 1.9; 1.1‐3.1:p = 0.019) were independent predictors of probable or possible dementia among participants in the study.

**Conclusion:**

This study highlights dementia as a growing public health issue in the African settings, in Uganda with over 1243 individuals affected in 2021. The findings emphasize the urgent need for investment in research and specialized services for older adults, particularly those with dementia.

